# Medical Association of Georgia

**Published:** 1877-05

**Authors:** 


### Editorial.

### MEDICAL ASSOCIATION OF GEORGIA.

This body held its annual meeting, for the present year, in Macon, on the 18th, 19th and 20th of April. The meeting was held in the same block where, about thirty years ago, the Association was organized with Dr. L. D. Ford, President.

The usual address of welcome was delivered by Dr. J. E. Blackshear, and was responded to by Dr. K. P. Moore.

About fifty members were in attendance, some half dozen or more of whom were admitted to membership at this meeting.

On the call for reports from sections, several papers were announced, by title, and afterwards read in regular order. Quite a number of volnntary contributions were also read during the meeting. Taken altogether, the number and quality of the papers presented indicate a lively interest felt by the profession in the Association and in the cause of medical science.

From the section of Practice of Medicine, Dr. W. R. Burgess—"Acute Rheumatism occurring two weeks after parturition, attended with suppuration. Recovery."

Dr. W. A. Green—"The physical and mental derangements resulting from the moral effects of the war."

Dr. G. F. Cooper—"Report of cases."

Dr. D. W. Hammond—"Elephantiasis"—"Lithotomy."

Dr. Charles P. Gordon—"Reminescences from the Case-Book of Memory and Surgical Report from 8th Congressional District."

Dr. J. G. Westmoreland—"Some evidences of Progress in Medicine." He also read a letter from Dr. J. T. Banks, in which mention is made of an epidemic of diphtheria that prevailed in Pike county; and of yellow fever in refugees from Savannah without communicating the disease in a single instance.

Dr. E. H. W. Hunter—"Report for 8th Congressional District."

From the Section of Gynæcology, Dr. LeHardy—"Report for 1st Congressional District."

Dr. A. J. Shaffer—"Report of two cases of Gynæcological Surgery."

From Section on Surgery, Dr. R. B. Doster—"Snag eight inches long piercing the abdomen. Extraction and recovery."

Dr. T. F. Walker—"Amputation for extensive necrosis of tibia"—"Gunshot wound with paralysis"—"Transverse Rupture of the Uterus."

Dr. A. W. Calhoun—"Successful transplanting conjunctiva of a rabit in the human eye for relief of deformity"—"Defects of hearing, etc., the result of enlarged tonsils requiring their excision."

Dr. R. M. Smith—"Ligation of the Primitive Carotid Artery."

Dr. A. J. Shaffer—"Report for 9th Congressional District with cases."

The voluntary contributions are as follows:

Dr. J. C. LeHardy—"The history, causes, nature, pathology and treatment of Yellow Fever, considering exclusively the epidemic of 1876, in Savannah." Also, "a successful case of Conservatine Surgery."

Dr. Jno. Thad. Johnson—"A comparison of several methods of treating Stricture of the Urethra."

Dr. Wm. Rawlings—"Case of Vaginal Tumor with Pathological Specimen."

The following verbal reports were made:

Dr. Robert Battey presented a modification of Atlee's uterine dilator, by Wilson, of Philadelphia, and spoke approvingly of it. He also alluded very favorably to the substitution of cinchonidia for quinia—uses it in same dose of quinine with satisfactory results.

Dr. J. H. Kenan reported a case of fractured tibia and fibula in an old man, who was the subject of epilepsy, successfully treated by plaster of Paris apparatus.

Dr. Wm. Abram Love presented a stem pessary of his own invention, made of copper, with a coating of zinc. The manner of retaining it in position, and its benefits were described.

Drs. Geo. F. Cooper, J. C. Johnson, and J. G. Westmoreland participated in a verbal discussion on the conditions necessary to the production of malaria.

At eleven o'clock on the second day the Annual Oration was delivered by Dr. J. S. Todd, of Atlanta, to an audience composed of citizens and members of the Association, on medical superstitions and the influence of the mind over the body. The subject is a very interesting one, and the address was made attractive by the felicitous style of the orator.

On motion of Dr. V. H. Taliaferro, Drs. W. A. Love, Jno. M. Johnson, Jas. F. Alexander, W. O'Daniel, and T. S. Hopkins, were appointed a committee to use their influence with the Legislature to have the laws in relation to the State Board of Health so amended as to render it practical and efficient. The committee was also directed to present this subject to the Constitutional Convention also.

A communication was received from Attorney General Ely, relative to the Board of Health.

The following by Dr. H. Smith, of Augusta, was adopted:

*Resolved*, That the special tax imposed upon physicians of this State is unequal and unjust, and that a committee be appointed to memorialize the Legislature to remove it.

The report of the committee on Inebriate Asylum was read, showing the failure to obtain favorable consideration by the Legislature.

Dr. Wm. O'Daniel resigned the office of Treasurer, and received a vote of thanks from the Association for his four years faithful service.

The following officers were elected on being reported by the nominating committee:

President, Wm. O'Daniel, of Twiggs; 1st. Vice President, D. W. Hammond, of Macon; 2d Vice President, S. B. Hawkins, of Americus; Secretary, J. B. Baird, of Atlanta; Treasurer, Wm. R. Burgess, of Macon.

Dr. C. H. Hall, of Macon, was appointed to the vacancy on Board of Censors.

Atlanta was selected as the place of meeting on third Wednesday in April, 1878.

We hope the committee of arrangements will make such ample and convenient preparations that the large attendance expected may be agreeably entertained.

